# Non-Invasive Imaging for Cardiovascular Interventions: An Evolving Paradigm

**DOI:** 10.5334/jbsr.1667

**Published:** 2018-12-05

**Authors:** Rodrigo Salgado

**Affiliations:** 1 Heilig Hartziekenhuis Lier, BE

**Keywords:** CT angiography, aortic stenosis, TAVI, TAVR

## Introduction

In the last decade, the medical community has seen a significant development in the treatment options for a range of cardiovascular diseases. Besides improved pharmacological options, advances in technology have allowed improved care by the use of, for example, new prosthetic cardiac valves in valvular heart disease and by further optimizing electrophysiological interventions in patients with selected rhythm disorders [1,2]. These new procedures have increasingly relied on non-invasive imaging primarily for pre-procedural planning in order to optimize procedural success and also with increasing potential for a more effective and comprehensive follow-up after an intervention. Besides echocardiography, computed tomography (CT) and to a lesser extent magnetic resonance (MR) imaging have evolved as an essential imaging tool in the selection of patients, avoidance of potential procedural hazards and early detection of complications. In this paper, we will focus on the role of CT in the pre- and post-procedural evaluation of transcatheter aorta heart valves (THV).

## Trancatheter Aortic Valve Replacement and the Role of the Radiologist

Aortic valve stenosis (AS) is the most common valvular heart disease in the elderly population [3]. It is a progressive disease, with severe AS having a 50% mortality rate only two years after onset of symptoms [4]. Once symptomatic, curative treatment is necessary to avoid progression to heart failure and death. Currently, the indicated treatment is surgical aortic valve replacement, restoring valvular function and prolonging survival compared to untreated subjects. Nevertheless, a subpopulation exists in which the surgical risks is aimed too high due to comorbidities and general frailty, leaving only palliative supportive care as the remaining option.

Recent developments in transcatheter-based therapies have provided an alternative therapeutic option for this patient subpopulation with critical aortic valve stenosis deemed inoperable or at high risk for surgery [5,6]. In this new approach, the native aortic valve is replaced by a bioprosthetic valve brought in place using a non-surgical endovascular or transapical pathway.

This new technique, called transcatheter aortic valve implantation (TAVI) or replacement (TAVR), not so long ago considered a merely theoretical possibility, has rapidly evolved into an accepted treatment method for the mentioned population. However, in order to achieve procedural success and limit complications, detailed knowledge of the anatomy of the aortic root and the potential access routes is necessary during the work-up of these patients.

While initial trials relied on echocardiography as the main imaging tool to assess the aortic root and annulus, its limitations as an operator-dependent and inherently two-dimensional imaging technique have led to an increased interest in the use of CT. CT, as a true 3D imaging modality, is better suited to correctly assess the complex anatomy of the aortic root and valve, allowing optimal anatomical assessment and further reduction of peri- and post-procedural complications. Therefore, an important task has as such been transferred to the radiologist to improve the work-up op potential TAVI candidates, allowing further refinement of the selection process of choosing the best valve size for the specific anatomy of the patient.

In order to properly assist the clinician or surgeon, a solid knowledge of the procedure, its implementation and the key anatomic elements is needed.

### The TAVR procedure

**Figure 1:**
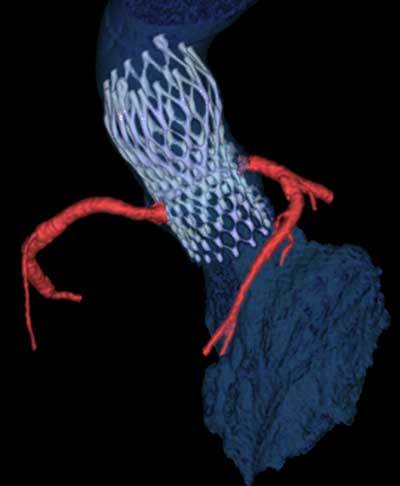
Self-expandable Corevalve transcatheter heart valve after deployment in the aortic root. The design of this valve has a component extending into the ascending aorta, which is a normal finding. As only the proximal portion of the THV at an annular and immediately supra-annular level is sealed, the coronary arteries (indicated in red) are not obstructed.

Fundamentally, a TAVR procedure consists of deploying a bioprosthetic aortic valve in the aortic root after transportation of the device from a chosen entry point. After deployment, this THV will functionally replace the diseased native valve, restoring valvular function (Figure 1). Placement can be retrograde, using the femoral or subclavian arteries as an endovascular access point, or anterograde percutaneous transapical through the apex of the left ventricle [2]. Finally, a suprasternal approach through the brachiocephalic trunk, an anterior approach through a minimal right anterior thoracotomy or a partial mini-sternotomy for transaortic placement through the ascending aorta is also possible [7].

**Figure 2:**
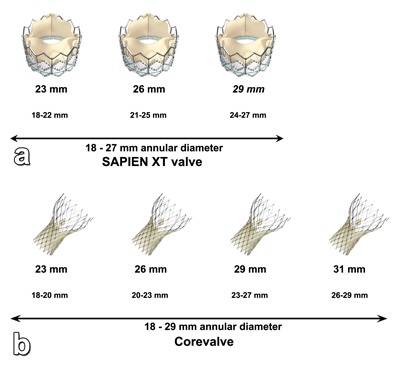
Available transcatheter valve sizes from Edwards Lifesciences **(a)** and Medtronic **(b)** with their corresponding ranges of aortic annular diameters. The diameter of the aortic annulus must be between 18 and 29 mm, regardless of other parameters. Correct pre-procedural measurement of the aortic annulus is an essential element to order to achieve maximal procedural success. As new models and devices are developed, the range of available sizes is likely to further expand in order to include as many potential TAVI candidates as possible.

Currently, two types of transcatheter valves are commercially available for TAVR. Edward Lifesciences (Edwards Lifesciences, Irvine, CA, USA) provides the balloon-expandable Sapien and Sapien XT valves. The latter valve is a slightly different version, introduced in Europe and featuring a cobalt-chromium lower profile frame. Medtronic produces the self-expandable CoreValve ReValving system and the newer Evolut series (Medtronic, Minneapolis, MN, USA). These transcatheter valves come in different sizes in order to accommodate as many potential TAVI candidates as possible (Figure 2). They also have different physical properties (Table 1), with further characteristics described in more detail elsewhere [2].

**Table 1:** Concise overview of the different properties of both Sapien and Corevalve transcatheter valves and their respective delivery systems.

	Sapien XT	CoreValve

Manufacturer	Edwards Lifesciences	Medtronic
Aortic annular range (mm)	18–27	18–29
Deployment	balloon-expandable	self-expandable
Frame	cobalt-chromium	nitinol
Pericardial leaflets	bovine	porcine
Valve function	intra-annular	supra-annular
Access routes	transfemoral transapical transaortic	transfemoral transaxillary transaortic
Delivery sheath size	22–24F 16/18F (Novaflex +)	18F

### Importance of correct anatomy assessment in the pre-operative evaluation

**Figure 3:**
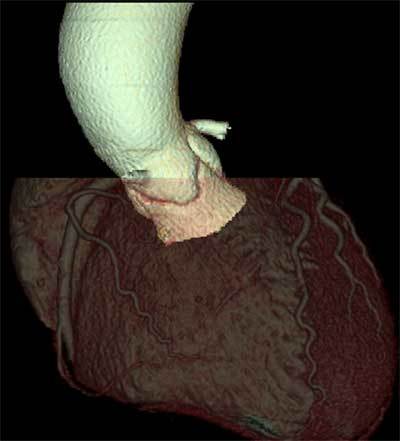
Volume-rendered CT image of the heart, indicating the double-oblique orientation of the aortic root and valve. As such, it is clear that a true 3D imaging modality like CT can provide additional benefits over CT in correctly assessing the aortic root anatomy and size in the pre-procedural TAVI work-up.

The aortic root has a rather central position in the heart, with a double-oblique orientation on 3D imaging (Figure 3). It extends from the left ventricular outflow tract to the sino-tubular junction, which marks the transition from the aortic sinuses (of Valsalva) to the tubular ascending aorta.

In contrast to these anatomic landmarks, the often-mentioned aortic annulus is not a real anatomic structure. It is a descriptive term often used by surgeons to refer to the insertion site of the aortic valve cusps, which is traditionally by many assumed to be always circular. The term aortic annulus is nevertheless anatomically not adequately defined, with a non-standard and variable interpretation. Consequently, some authors have suggested refraining from using this term. Its use is nevertheless widespread and deeply embedded in scientific literature and communications.

**Figure 4:**
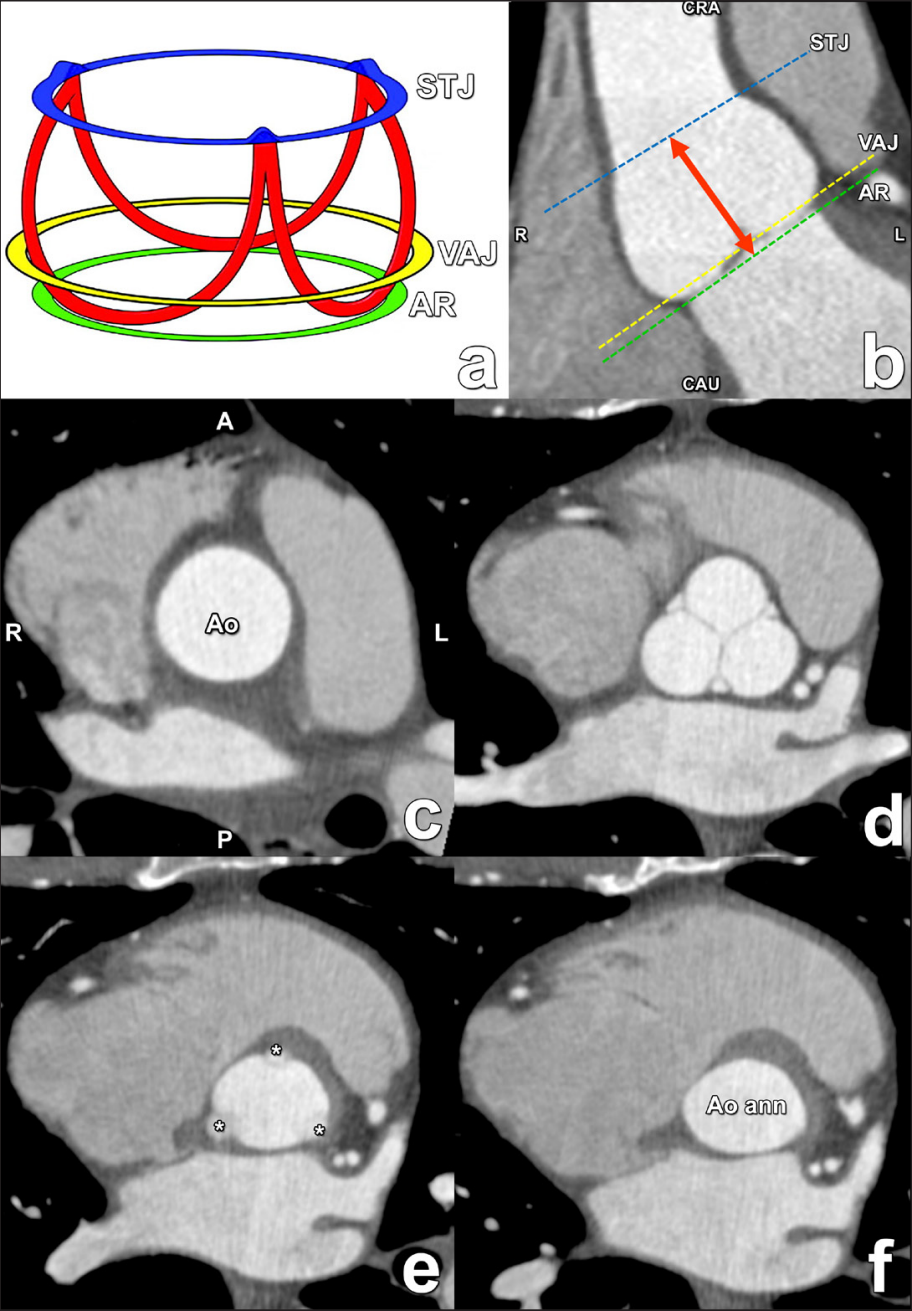
Drawing illustrating the crownlike suspension of the aortic valve leaflets within the aortic root extending across the length of the aortic sinus **(a)**. AR = virtual annular ring (green), formed by joining the basal attachments of the aortic valve leaflets; STJ = sinotubular junction (blue); VAJ = ventriculoarterial junction (yellow). Red = aortic leaflet insertion sites in the sinus of Valsalva forming a crownlike ring. **(b)** Coronal contrast-enhanced CT image demonstrates the levels of the sinotubular junction (STJ) (blue line), ventriculoarterial junction (VAJ) (yellow line), and annular ring (AR) (green line). Double-headed arrow = anatomic range of the sinuses of Valsalva. CAU = caudal, CRA = cranial. **(c–f)** Double-oblique reformatted images further clarify the changing shape of the aortic root contour. (c) The sinotubular junction forms the top of the crown, where the outlet of the aortic root in the ascending aorta (Ao) is a true circle. A = anterior, P = posterior. (d) The aortic root gradually becomes less circular, with a more cloverleaf shape at its midportion (i.e., at the sinuses of Valsalva). At this level, the aortic valve leaflets are clearly seen. (e) The aortic valve leaflets (*) are just barely visible at the level of the ventriculoarterial junction, where the left ventricular structures give rise to the fibroelastic walls of the aortic valvar sinuses. Note that the aortic root contour is now becoming increasingly ellipsoid. (f) The bottom of the aortic root is formed by the virtual ring, or aortic annulus (Ao ann), which has an oval shape in most patients.

In practice, the term ‘annulus’ is commonly used to specifically indicate a virtual ring formed by the nadir of the attachment sites of the aortic valve leaflets. This attachment of the aortic valve leaflets to the aortic root wall is not circular: it has a more semilunar, crown-shaped morphology extending from the sino-tubular junction to the basal attachment plane of the aortic valve leaflets (the so-called annulus), located just below the ventriculo-arterial junction (Figure 4). Given its conical form, the annulus is perhaps more appropriately described as a three-pronged coronet rather than a ring.

**Figure 5:**
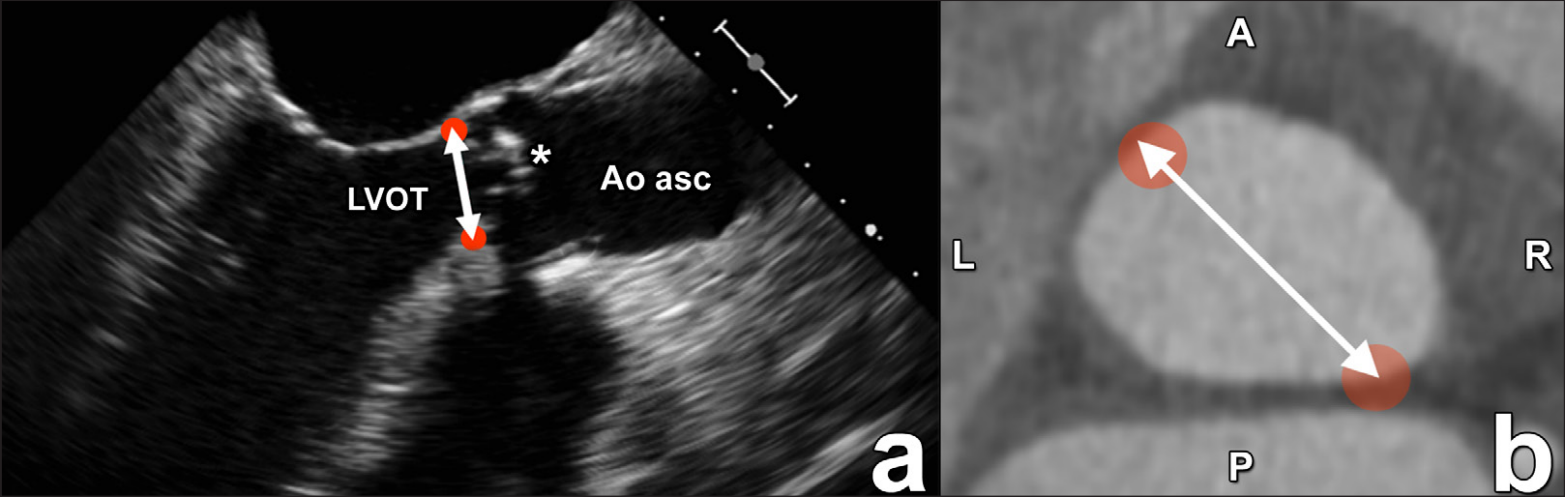
Illustration of the difficult correlation of echocardiography-based versus CT-based measurement of the aortic annulus. Being a two-dimensional imaging modality, echocardiography provides only one measurement as the largest annular diameter, as perceived by the performing physician or technologist. In contrast, it is now generally accepted that the cross-sectional diameter of the annulus on 3D imaging modalities like CT is mostly oval, providing a short and a long axis. Therefore, CT measurements and echocardiography measurements of the aortic root and annulus cannot be easily compared.

With transcatheter aortic valve replacement, unlike surgical valve replacement, annular sizing is not performed under direct inspection but rather on the basis of non-invasive imaging. Imaging guidance is needed to ensure the appropriate degree of oversizing to help avoid paravalvular regurgitation and yet prevent excessive oversizing to help reduce the potential risk of annular rupture. It is here that CT provides added value over echocardiography, correctly assessing the often oval morphology and different sizing parameters of the aortic annulus (Figure 5).

The final selection of access route will depend on the combination of the chosen device, the physical properties of corresponding delivery systems and the adequacy of the investigated pathway. A carefully chosen access route is therefore one of the key components for procedural eligibility and success, since in an individual case different pathways can be associated with a potentially different risk for peri- and post-procedural vascular and embolic cerebrovascular complications. This emphasizes the need for an individually optimized access route selection.

**Figure 6:**
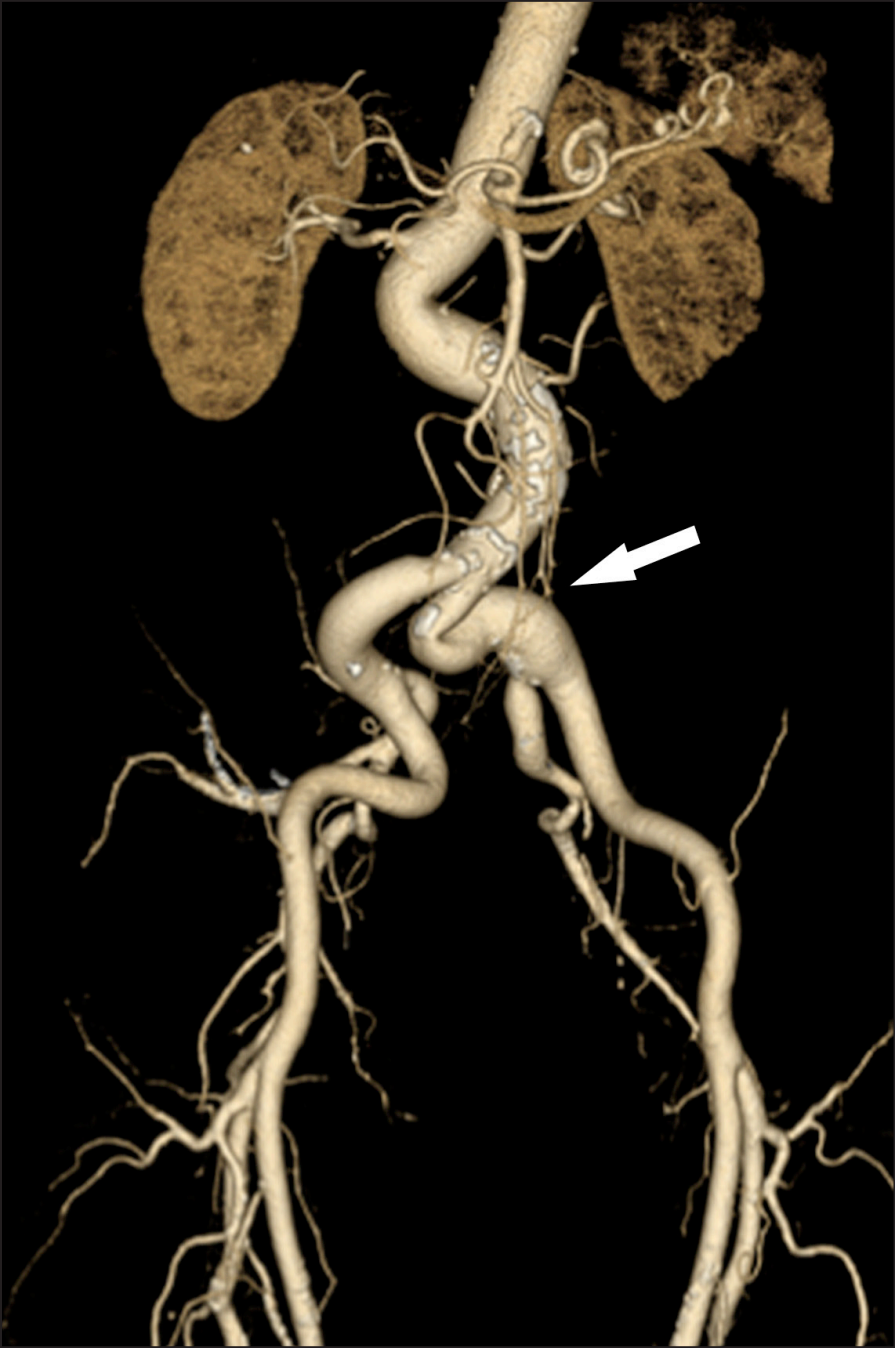
Inadequate access route patency. Volume-rendered CT image obtained in a 74-year-old patient shows general tortuosity of the aortoiliac arterial vessels, a finding that is most prominent in the left common iliac artery (arrow). This pronounced tortuosity will increase procedural risk, in this case making the right iliac arteries a better (although not perfect) option.

Both device- and anatomy-related obstacles may alter the chosen access path or make the procedure through the endovascular pathway even technically impossible regardless of device-compatible aortic root dimensions (Figure 6). Therefore, MDCT has an important role in examining the potential access routes and to report any possible problems which may alter the chosen access strategy [2].

An overview of all required pre-procedural information is given in Table 2.

**Table 2:** Required information from pre-procedural CT examinations.

Anatomic level	Comment

Aortic annulus	Provide preferential systolic measurements if available. Include annular cross-sectional diameters (long- and short axis), circumference and annular area. Also calculate circumference- and area-derived diameter. Determine recommended fluoroscopic projection angle for orthogonal view on annular plane (if not determined during procedure by, e.g., three-dimensional rotational angiography).
Aortic valve (native)	Comment (descriptive) on extent and distribution of valve calcifications. Cuspidity (bicuspid, tricuspid, other variants).
Aortic root	Provide shortest distance from the aortic annulus to the ostia of the left main artery and the right coronary artery.
Left atrium	Exclude a left atrial appendage thrombus, either with transesophageal echocardiography or with delayed phase imaging.
Left ventricle	Evaluate the presence of basal septal hypertrophy and wall thrombi.
Ascending aorta	Comment on extent of wall calcifications (especially if transaortic approach is considered), and presence of atherosclerotic thrombi. Comment on the angulation between the ascending aorta and the LVOT.
Aortic arch, descending aorta and abdominal aorta	Branch anatomy of aortic arch (for subclavian approach). Descriptive assessment of atherosclerosis, including tortuosity, intraluminal obstruction and thrombi.
Subclavian arteries and brachiocephalic artery	Report on patency and luminal diameter (left subclavian artery is preferred).
Common and external iliac arteries and common femoral artery	Report on patency and luminal diameter. Descriptive assessment of atherosclerosis including tortuosity. Report any potential problems at the targeted femoral punction site (e.g., pre-existing pseudo-aneurysm).

### Paravalvular leakage and the importance of sizing

One of the most prognostically significant complication after the procedure is the occurrence of a paravalvular leak (PVL). In such an event, there is usually inadequate deployment of the prosthetic valve in the aortic root, allowing passage of blood between the transcatheter valve and the aortic wall. This leakage not only compromises valvular function, but also has been shown to be a significant marker for post-procedural survival. Even moderate leakage, once considered of minor significance, has been shown to significantly impact survival.

**Figure 7:**
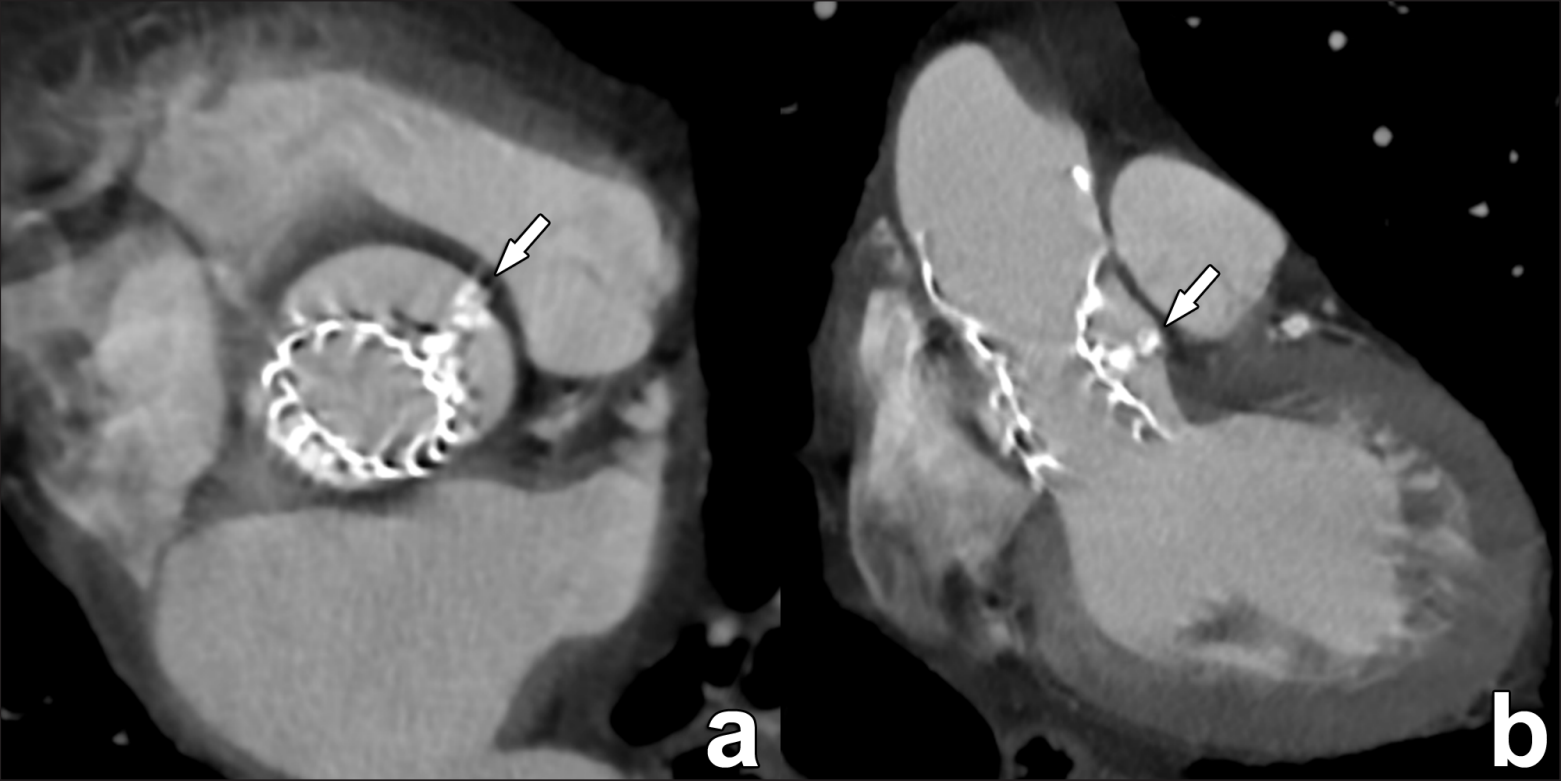
CoreValve device asymmetry caused by extensive calcifications. Coronal in-plane double-oblique **(a)** and slightly oblique **(b)** CT images reveal prominent displaced calcifications (arrow) between the transcatheter valve and the aortic root wall, leading to incomplete deployment and an asymmetric position of the prosthesis in the aortic sinus. Although these images are very convincing for a large paravalvular leak, several echocardiographic examinations revealed only moderate leakage with no evident clinical consequences. This case illustrates the strength of echocardiography in providing both anatomic and functional information, making it the standard of reference for the evaluation of paravalvular leakage.

While many factors may contribute to the presence of PVL, one of the parameters is the incorrect sizing of the transcatheter valve to the native anatomy. In particular, a smaller valve size will increase the likelihood of incomplete adherence to the aortic wall, increasing the risk of PVL. Also, THV deployment may also be compromised by prominent native leaflet calcifications. As these calcified leaflets are crushed between the deploying THV and the aortic wall, they may lead to regional incomplete valve expansion (Figure 7).

The incorporation of a true 3D imaging modality like CT has further refined the valve selection process, not only by identifying eligible patients whose annular anatomy falls within the size ranges of available devices, but also in assisting in choosing the best valve size leading to optimal patient–valve size matching.

### Post-operative evaluation

While CT, as a morphology-based imaging modality, cannot reliably detect PVL, it can be used in the work-up of a patient with transcatheter valve dysfunction. Besides evaluating the position and expansion of the THV, it can also detect prosthetic valve thrombosis. Several studies have shown that CT can detect early leaflet thickening, thrombus and pannus with a higher sensitivity than ultrasound [8,9]. While it can as such provide important information in the evaluation of a patient with prosthetic valve dysfunction, the clinical significance of discrete thrombotic leaflet thickening as detected with CT in patients with no clinical symptoms and normal valve function has not yet been determined.

Despite the relative recent appearance of TAVR, a worldwide effort has led to a vast amount of research primarily focusing on the role of CT in the pre-procedural setting. While the role of CT in the post-procedural setting is currently less clearly defined, new insights have provided fresh opportunities to further improve clinical care by integrating CT in the clinical routine. One of the currently most debated topics is the presence of thrombus in prosthetic heart valves of various types, in which CT has proven itself to be a very useful tool for detecting both valvular thrombus and post-operative pannus. Given the clinical impact of such complications and the development of transcatheter repair or replace techniques for a diseased mitral valve, one can expect that the role of CT in the management of prosthetic heart valves will only increase.

While an extensive review of the post-procedural findings and the most common complications is beyond the scope of this paper, some restraint is indicated when trying to extract functional conclusions from anatomical findings. Currently, echocardiography remains after the procedure the single most used imaging tool, providing bedside functional information at a relatively low cost. While studies regarding potential CT-derived anatomical markers that can be correlated with functional findings are appearing, the current position of echocardiography as a primary source of functional information is unlikely to be challenged soon. Also, MR imaging, which can provide anatomical and functional information in a single examination, seems intrinsically well suited as a problem-solving tool in unequivocal cases. Therefore, multimodality imaging of prosthetic heart valves will further develop using the strengths of both CT and MR.

### Future Perspectives

As results of large trials are being processed, recent evidence suggests that TAVI could also be safely applied in individuals with an intermediate surgical risk [4,10,11]. Also, TAVI has also been successfully been implemented in patients with bicuspid valves [12,13]. This is important, as this type of congenital valve type is more frequent in younger patients who currently undergo surgical valve replacement when indicated. Nevertheless, one the remaining question is the long-term durability of the used THV so far. Given the relative recent implementation of TAVI, more data here is needed before this technique can be safely implemented.

The worldwide success of THV for aortic valve replacement has generated intense further research in the refinement of current valve models, the development of new generations of valves, and the exploration of transcatheter techniques for other cardiac valves. In recent year there have been numerous publications from trials exploring the options for transcatheter mitral valve repair and even replacement [14,15]. Also, in these applications, CT is confirming its important role as the most adequate 3D imaging investigation to evaluate the relevant anatomy, providing essential information to achieve maximum procedural success.

## Conclusion

It is clear that the role of the cardiovascular radiologist will only gain in importance as new treatment options emerge and precise imaging-derived pre- and post-procedural information is required.

Radiology is a medical specialty by nature especially susceptible to technology advances. Some of these innovations have led to significant changes in clinical care. At the very least, these developments provide an opportunity to place the radiologist back in the clinical decision-making process, becoming as such more involved in actual patient care and by extension improving its undeserved reputation as a merely technology-driven specialist detached from clinical care. The importance of this cannot be overstated, as turf wars have emerged at the crossroads of medical specialties. By consistently underscoring our added value, this approach may provide the best professional defence, and the best way to achieve clinical success.

## References

[1] Salgado, R and Ghaye, B. Electrophysiology interventions In: Dewey, M (ed.), Cardiac CT. 2015; 498 Heidelberg: Sprinfer-Verlag.

[2] Salgado, RA, Leipsic, JA, Shivalkar, B, et al. Preprocedural CT evaluation of transcatheter aortic valve replacement: What the radiologist needs to know. Radiographics. 2014; 34(6): 1491–514. DOI: 10.1148/rg.346125076 25310413

[3] Osnabrugge, RL, Mylotte, D, Head, SJ, et al. Aortic stenosis in the elderly: Disease prevalence and number of candidates for transcatheter aortic valve replacement: A meta-analysis and modeling study. J Am Coll Cardiol. 2013; 62(11): 1002–12. DOI: 10.1016/j.jacc.2013.05.015 23727214

[4] Baumgartner, H, Falk, V, Bax, JJ, et al. ESC/EACTS Guidelines for the management of valvular heart disease. Eur Heart J. 2017; 38(36): 2739–91. DOI: 10.1093/eurheartj/ehx391 28886619

[5] Leon, MB, Smith, CR, Mack, M, et al. Transcatheter aortic-valve implantation for aortic stenosis in patients who cannot undergo surgery. N Engl J Med. 2010; 363(17): 1597–607. DOI: 10.1056/NEJMoa1008232 20961243

[6] Smith, CR, Leon, MB, Mack, MJ, et al. Transcatheter versus surgical aortic-valve replacement in high-risk patients. N Engl J Med. 2011; 364(23): 2187–98. DOI: 10.1056/NEJMoa1103510 21639811

[7] Philipsen, TE, Collas, VM, Rodrigus, IE, et al. Brachiocephalic artery access in transcatheter aortic valve implantation: A valuable alternative: 3-year institutional experience. Interact Cardiovasc Thorac Surg. 2015; 21(6): 734–40. DOI: 10.1093/icvts/ivv262 26395943

[8] Salgado, RA, Budde, RP, Leiner, T, et al. Transcatheter aortic valve replacement: Postoperative CT findings of Sapien and CoreValve transcatheter heart valves. Radiographics. 2014; 34(6): 1517–36. DOI: 10.1148/rg.346130149 25310415

[9] Makkar, RR, Fontana, G, Jilaihawi, H, et al. Possible subclinical leaflet thrombosis in bioprosthetic aortic valves. N Engl J Med. 2015; 373(21): 2015–24. DOI: 10.1056/NEJMoa1509233 26436963

[10] Leon, MB, Smith, CR, Mack, MJ, et al. Transcatheter or surgical aortic-valve replacement in intermediate-risk patients. N Engl J Med. 2016; 374(17): 1609–20. DOI: 10.1056/NEJMoa1514616 27040324

[11] Reardon, MJ, Van Mieghem, NM, Popma, JJ, et al. Surgical or transcatheter aortic-valve replacement in intermediate-risk patients. N Engl J Med. 2017; 376(14): 1321–31. DOI: 10.1056/NEJMoa1700456 28304219

[12] Jilaihawi, H, Chen, M, Webb, J, et al. A bicuspid aortic valve imaging classification for the TAVR era. JACC Cardiovasc Imaging. 2016; 9(10): 1145–58. DOI: 10.1016/j.jcmg.2015.12.022 27372022

[13] Perlman, GY, Blanke, P and Webb, JG. Transcatheter aortic valve implantation in bicuspid aortic valve stenosis. EuroIntervention: Journal of EuroPCR in collaboration with the Working Group on Interventional Cardiology of the European Society of Cardiology. 2016; 12(Y): Y42–5. DOI: 10.4244/EIJV12SYA10. 27640030

[14] Blanke, P, Dvir, D, Cheung, A, et al. Mitral annular evaluation with CT in the context of transcatheter mitral valve replacement. JACC Cardiovasc Imaging. 2015; 8(5): 612–5. DOI: 10.1016/j.jcmg.2014.07.028 25937198

[15] Blanke, P, Naoum, C, Webb, J, et al. Multimodality imaging in the context of transcatheter mitral valve replacement: Establishing consensus among modalities and disciplines. JACC Cardiovasc Imaging. 2015; 8(10): 1191–208. DOI: 10.1016/j.jcmg.2015.08.004 26481845

